# G6PD deficiency—does it alter the course of COVID-19 infections?

**DOI:** 10.1007/s00277-023-05164-y

**Published:** 2023-03-11

**Authors:** Tsz Yuen Au, Oskar Wojciech Wiśniewski, Shamiram Benjamin, Tadeusz Kubicki, Dominik Dytfeld, Lidia Gil

**Affiliations:** 1 Faculty of Medicine, Poznan University of Medical Sciences, Poznan, Poland; 2 Department of Hematology and Bone Marrow Transplantation, Poznan University of Medical Sciences, Poznan, Poland

**Keywords:** COVID-19, G6PD deficiency, Oxidative stress, Antioxidative therapy, Viral load

## Abstract

Despite the existence of well-founded data around the relationship between reactive oxygen species (ROS) and glucose-6-phosphate dehydrogenase (G6PD), current research around G6PD-deficient patients with viral infections, and limitations as a result of their condition, are inadequate. Here, we analyze existing data around immunological risks, complications, and consequences of this disease, particularly in relation to COVID-19 infections and treatment. The relationship between G6PD deficiency and elevated ROS leading to increased viral load suggests that these patients may confer heightened infectivity. Additionally, worsened prognoses and more severe complications of infection may be realized in class I G6PD-deficient individuals. Though more research is demanded on the topic, preliminary studies suggest that antioxidative therapy which reduces ROS levels in these patients could prove beneficial in the treatment of viral infections in G6PD-deficient individuals.

## Introduction

Glucose-6-phosphate dehydrogenase (G6PD) is an enzyme responsible for reducing NADP^+^ to NADPH via oxidation byglucose-6 phosphate. Produced NADPH is then utilized by glutathione reductase to reduce oxidized glutathione back into its active state. Thereby, the reduced glutathione works as an antioxidant to neutralize reactive oxidative species (ROS) via electron donation [1]. The production of ROS, such as hydrogen peroxide or superoxide and hydroxyl radicals, is an integral part of human metabolism. Moreover, inflammation or adverse medication effects may lead to elevated ROS within organisms [1, 2]. G6PD deficiency is one of the most common enzymopathies with more than 400 million individuals affected worldwide, mostly men [3]; it is an X-linked recessive genetic disorder characterized by the markedly reduced enzymatic activity of G6PD as a result of defective production. Low G6PD levels lead to an increase in oxidized and non-functional glutathione. The consequent ROS accumulation in the body is inevitably associated with tissue injury [1], since ROS are known to damage cell components, particularly DNA, leading to loss of function, induction of apoptosis, or even carcinogenesis [2]. Most patients with G6PD deficiency are asymptomatic until exposed to environmental triggers that increase ROS production; potential triggers include viral infections, certain foods—notoriously fava beans—or various medications: sulfa drugs or particular antibiotics [4]. Following trigger exposure, hemolysis, anemia, jaundice, or even renal failure may be classically present, though occurring symptoms may differ between variants (Table 1). Regardless of variant, ROS-mediated erythrocytic hemolysis may drive the patient into an anemic state. Furthermore, hemoglobin damaged by ROS may accumulate and form Heinz bodies within erythrocytes [1, 5].

**Table 1:** Classification of G6PD deficiency “WHO classification” [79, 80]

Class	Previous Criteria	Reviewed Criteria
I	less than 10% of standard G6PD activity. Severe G6PD deficiency with CNSHA	Severe G6PD deficiency associated with CNSHA
II	less than 10% of standard G6PD activity. Severe G6PD deficiency	less than 10% of standard G6PD activity. Severe G6PD deficiency
III	10 to 60% of standard G6PD activity. Moderate to mild enzyme deficiency with intermittent acute hemolysis	10 to 60% of standard G6PD activity. Moderately G6PD deficiency
IV	60% to 100% of standard G6PD activity. Very mild or no enzyme deficiency	60% to 150% of standard G6PD activity. Normal G6PD activity
V	More than twice of normal G6PD activity. Overactive G6PD	Increased G6PD activity

*CNSHA* chronic non-spherocytic hemolytic anemia; *GGPD* glucose-6-phosphate dehydrogenase

An early in vitro study has illustrated that a deficiency in G6PD may increase human susceptibility to infection by coronavirus 229E [6]. Though similar studies on severe acute respiratory syndrome coronavirus 2 (SARS CoV-2) have not yet been performed, due to existing similarities in these pathogens and the immunological response they trigger, recent studies have suggested that G6PD deficiency may potentially impact the prognosis, clinical outcomes, and severity of SARS-CoV-2 infection; this may be due to the nature of the condition or via the limitation of therapeutic options available to these patients [7–12]. Elevated levels of oxidative stress as a result of G6PD deficiency cultivate a favorable environment for viral replication, consequently deteriorating the course of infection [12]. Additionally, the activity of neutrophils, cytokines, and inflammasomes is typically impaired in those with G6PD deficiency, which may cause increased susceptibility to coronavirus disease 2019 (COVID-19) and other viral infections [13–18]. Furthermore, hydroxychloroquine, a medication previously used to treat COVID-19 despite being rapidly abandoned as a therapeutic option, increases oxidative stress in patients which may incidentally trigger hemolytic anemia; this effect may drastically exacerbate symptoms in patients with G6PD deficiency. Current FDA recommendations suggest remdesivir as a treatment for COVID-19 patients; however, those with G6PD deficiency may exhibit more severe side effects than those with in-tact G6PD as a result of a decreased threshold for processing ROS in the liver. These examples illustrate some limitations of pharmacological agents which may be utilized in treating COVID-19 among this demographic of patients.

Altogether, the aim of this paper is to review existing literature regarding the impact of G6PD deficiency on the course and management of COVID-19 infections. In the following sections of the manuscript, we emphasize the role of immunologic disturbances associated with G6PD deficiency, such as neutrophil dysfunction, impaired inflammasome activation, and disruption in the NF-κB signaling pathway, which may contribute to increased ROS production, viral replication, and contagiousness in SARS-CoV-2 infection. Furthermore, we pay attention to COVID-19 complications potentially associated with ROS accumulation, including hemolysis, thrombosis, and elevated cardiovascular risk. Moreover, we show limitations on COVID-19 treatment in G6PD-deficient individuals resulting from the pathophysiological background of the underlying disease. Finally, we discuss current findings and suggest directions for further research regarding the relationship between COVID-19 and G6PD deficiency.

## Impaired immune response against COVID-19 in G6PD-deficient patients.

Recent studies have suggested that G6PD plays an important role in immune response and that G6PD deficiency may increase susceptibility to infections [12, 16]. Neutrophils are the most abundant leukocytes in the system and play a major role in the innate immune response. They are the first cells to arrive at the infection site and are responsible for the neutralization of pathogens and the recruitment of additional immune cells. Although the role of neutrophils in bacterial infection is well understood, the mechanism of the neutrophilic response in viral infection has not yet been extensively studied [15, 19, 20]. There are several studies suggesting that neutrophils have the ability to phagocytose various viruses, including *Influenza*, *Cytomegalovirus,* and *Herpes simplex* viruses [20–24]. However, present understanding regarding the role of neutrophils in COVID-19 infection is notably limited.

Neutrophil extracellular traps (NETs) are comprised of modified neutrophilic chromatin that is expelled into the system to neutralize and prevent the dissemination of microbes, whilst concurrently alerting the immune system of the infection [25–27]. Early studies have confirmed that G6PD-deficient patients may display impeded neutrophilic function and impaired NETs, potentially affecting the immune system’s ability to clear infections [15–18]. Studies and case reports have revealed that NET formation may increase dramatically in COVID-19 infections, suggesting that neutrophils and NETs play a substantial role in immunity against SARS-CoV-2 [28, 29]. Within these studies, increased NET formation was also specifically associated with certain complications of COVID-19 infections, including vascular occlusion and pneumocyte damage, allowing NETs to be labelled as multi-purposed in the immune response against SARS-CoV-2 and other viruses [26, 28–31].

Interleukin-1β (IL-1β) is a mediator of immunity produced by monocytes and macrophages during infection and is essential to the host response against pathogens [32, 33]. Studies have shown that IL-1β and NOD-, LRR- and pyrin domain-containing protein 3 (NLRP3) inflammasome activation is crucial in inhibiting viral replication via maintenance of optimal interferon and immune response [34, 35]. The disruption of IL-1β and NLRP3 can lead to impairment of the innate cellular immune response, which may have varying clinical implications during infections [13]. IL-1β binds to the interleukin-1 receptor (IL-1R) to activate myeloid differentiation primary response 88 (MyD88) and nuclear factor kappa-light-chain-enhancer of activated B cells (NF-κB); these upregulate the expression of genes specific for immune-mediated inflammation, adaptive immunity, and antiviral response [33, 34]. Type I Interferons (IFNs) and IL-1β are known to work together to inhibit viral replication [34, 35]. One study supports that the absence of IL-1R signaling results in a reduction of type I IFNs, thus leading to an increase in viral load and potentially increasing host mortality rates as well [34]. Furthermore, studies suggest that the reduction of IL-1β and inhibition of the NLRP3 inflammasome could impair the anti-viral immune response, exposing patients with IL-1β and NLRP3 inhibition to a more severe course of a viral infection than those without. A recent study revealed a significant decrease in IL-1β expression and defective NLRP3 inflammasome activation in G6PD-deficient patients [13]. This supports that G6PD-deficient patients may be more susceptible to viral and bacterial infections, which may also encompass COVID-19 infections. Elevated levels of IL-1β and the activation of NLRP3 inflammasome were observed in SARS-CoV-2 infected patients [36, 37], implying that they are involved in immunologic defence mechanisms against COVID-19 infection.

## Elevated ROS in G6PD-deficient patients favors viral replication.

**Fig. 1:**
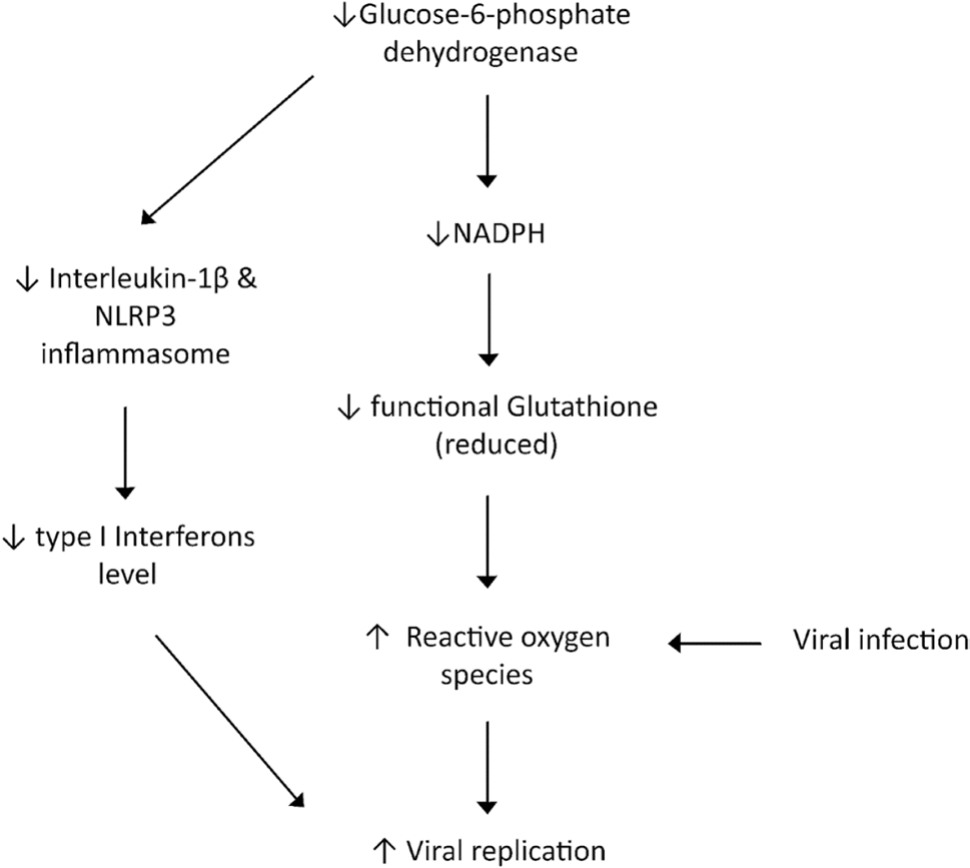
Pathways that increase viral replication [1, 6, 13, 38, 41, 42]. The diagram iterates the suggested pathways that could lead to increased viral replication in G6PD deficiency. Interleukin-1β and NLRP3 inflammasome were found to be impaired in G6PD deficiency, which results in impeded type 1 interferons level. The low level of G6PD enzymes and viral infection gives rise to the accumulation of ROS. Both pathways favor viral replication

It has been shown that viral infections may trigger NF-κB activation, leading to the inhibition of viral replication [6, 38]; the NF-κB signaling pathway is also involved in regulating oxidative stress in the human body [39, 40]. However, in G6PD-deficient cells, the ability to activate any NF-κB-mediated immune response is impaired due to an imbalance in reduction and oxidation mechanisms (Fig. 1) [38]. In these cells, an increased viral load was detected with concomitant ROS elevation. Meanwhile, the subsequent introduction of antioxidant agents like lipoic acid led to the alleviation of these conditions [6, 41, 42]. The results of these in vitro studies suggest that elevated ROS facilitates the replication process in an array of viruses, including coronaviruses, especially in G6PD-deficient cells where the level of ROS is dysregulated [6, 38, 41, 42]. As long as the in vitro studies translate to human physiology, these results further support the positive feedback loop between ROS concentration and viral load, resulting in a higher risk of severe infection in G6PD-deficient patients. This increased viral load can therefore be potentially attributed to the absence of specific and efficient antiviral medication targeting SARS-CoV-2. Consequently, the management of ROS levels seems to be beneficial in the treatment of COVID-19 patients. If an increased viral load corresponds with increased infectivity, COVID-19 patients with G6PD deficiency could therefore be more contagious than patients with in-tact G6PD enzymes [43].

## Risk of cardiovascular and hemolytic disease in G6PD-deficient patients.

It is well understood that patients with G6PD deficiency are more prone to thrombotic and hemolytic events [10]. To elucidate this concept, clinical cases have revealed that COVID-19 infection in G6PD-deficient patients could further elevate the risk of such events, leading to more severe clinical outcomes such as intravascular and extravascular hemolysis [1, 10, 44, 45]. In G6PD-deficient patients, acute hemolysis occurs when an elevation of oxidative stress is triggered by viral infection, certain medications, or even fava beans. Typically, increased NADPH, which reduces glutathione, is able to compensate for elevated levels of oxidative stress caused by such triggers. Nevertheless, the presence of impaired G6PD activity may result in ROS accumulation, causing severe hemolysis; Fig. 1 illustrates how G6PD concentration correlates with ROS levels. When ROS levels exceed the metabolic capacity of active glutathione, cell damage, thrombosis, and acute hemolytic anemia may occur; concomitant viral infection may exacerbate existing symptoms of infection and cause the failure of multiple organs [3, 9, 10, 46]. Moreover, this condition could be further exacerbated in G6PD-deficient patients who are elderly, as increased age is correlated with reduced G6PD expression [14].

An increased incidence of venous thromboembolism in patients with G6PD deficiency has been addressed in previous literature, attributed to various potential causes [45, 47]. Factor V Leiden is a prothrombotic condition where the degradation of clotting factor V is impaired, resulting in an increased risk of venous thrombosis [47]. One study report that Factor V Leiden is present in 11% of G6PD-deficient patients, a significantly higher incidence than the 2.4% reported in the normal population of Dalmatia [47].

Additionally, the advanced progression and rupture of atherosclerotic plaques may contribute to an increased incidence of thrombosis. The combination of low NADPH concentration and high oxidative stress may lead to the progression and pathogenesis of atherosclerosis [48]. Higher levels of ROS and inflammation within atherosclerotic lesions favor the loss of collagen and suppress its production; this is associated with thinning of the fibrous cap leading to decreased stability of atherosclerotic plaques and an increased risk of plaque rupture [49]. Rupture of atherosclerotic plaques is inherently associated with endothelial disruption, one element of the Virchow Triad, and thereby one of three main factors triggering thrombosis; static blood flow and hypercoagulable states like Factor V Leiden comprise the other two elements [49, 50]. Thus, elevated oxidative stress does not merely accelerate the progression of atherosclerosis, but can also increase the risk of plaque rupture, which may directly lead to a thrombotic event.

In addition to an increased incidence of hemolytic and thrombotic events, increased risk of cardiovascular disease development has been observed in previous studies; this includes coronary heart disease, cerebrovascular disease, peripheral arterial disease [51, 52]. One study specifically identified the infection as a risk factor of cardiovascular disease development in elderly G6PD-deficient patients [51]. Although the study focused on a specific bacterial infection, it is fair to assume that other pathogens may elicit a similar response [51]. Further studies could be done to evaluate this assumption. Moreover, it is consequently logical to think that G6PD-deficient patients are more prone to COVID-19-induced myocardial injury and other cardiovascular complications [48]. The aforementioned impaired ability to neutralize ROS may also increase the risk of reperfusion injury in this patient population. Therefore, continual monitoring of cardiac function in early stages of infection could prove beneficial in patients with G6PD deficiency.

## Limitations on COVID-19 treatment in G6PD-deficient patients.

Historically, chloroquine and hydroxychloroquine have been utilized to induce oxidative stress in order to kill malarial parasites [9, 10, 53]. However, at the start of the COVID-19 pandemic, both drugs were used to treat COVID-19 infections due to their ability to increase endosomal pH, inhibiting both the fusion of SARS-CoV-2 and the angiotensin-converting enzyme 2 (ACE2) receptor presented on the host cell membrane [54, 55]. In either case, the mechanism of these drugs cause increased systemic oxidative stress; this fairly establishes their use as contraindicated in patients with G6PD deficiency [4]. In multiple case reports, hydroxychloroquine administration for the treatment of COVID-19 in G6PD-deficient patients revealed a dramatic drop in hemoglobin and haptoglobin, indicating erythrocyte breakdown [56–60]. Since COVID-19 infection in patients with G6PD may independently promote hemolysis, prescribing hydroxychloroquine or chloroquine may exacerbate this hemolytic effect as a result of increased oxidative stress. Although recent studies have proven chloroquine and hydroxychloroquine inefficient in treating COVID-19, it is still worthwhile to evaluate whether these drugs are safe for G6PD-deficient patients [61].

Currently, according to the Food and Drug Administration (FDA), remdesivir is the first medication to be approved for the treatment of COVID-19 [61]. Remdesivir is a nucleoside analog that inhibits the RNA-dependent RNA polymerase (RdRp) of SARS-CoV-2, which results in impaired viral replication [62]. Since adverse hepatotoxic effects are common with remdesivir, severe impairment of hepatic function is a strict contraindication of its use [63, 64]. In G6PD-deficient patients, liver vulnerability is often expected due to the low concentration of G6PD enzymes and high oxidative stress in hepatocytes. It has been shown that liver enzymes, including alanine transaminase (ALT) and aspartate transaminase (AST), are significantly higher in G6PD-deficient patients than in unaffected individuals [65]. This finding supports the idea that the liver in G6PD-deficient patients is more susceptible to damage and drug-related toxicity; therefore, medications with hepatotoxic effects could prove injurious to the already vulnerable G6PD-deficient liver. At present, there are no clinical studies evaluating the impact of medication use in G6PD-deficient patients. Thus, the safety of remdesivir utilization in G6PD-deficient subpopulations needs to be elucidated and requires further research. Additionally, other antiviral medications, such as molnupiravir and Paxlovid (combination of nirmatrelvir and ritonavir), were evaluated and authorized for emergency use in the treatment of COVID-19 [66–68]. Given the immense potential benefit to G6PD-deficient patients if new and safe treatment options arise, rigorous clinical studies must be performed to determine the safest choice of drugs for these patients.

## Recent studies and directions for further research

Despite an array of laboratory findings suggesting that COVID-19 patients with G6PD deficiency may suffer a worse prognosis, one clinical study concluded that G6PD-deficient patients might experience less severe symptoms, requiring reduced ventilatory support and an overall lower case-fatality rate than patients with in-tact G6PD [11]. This could be potentially explained by the nature of the G6PD enzyme. In addition to its well-known antioxidative activity, G6PD exhibits a pro-inflammatory mechanism of action [69]; activated G6PD may enhance oxidative inflammation in acute lung injury during infection, possibly exacerbating clinical symptoms [70]. It is understood that complications due to aggressive host immune response—such as dramatic apoptosis as a result of fulminant inflammation in lungs and other organs—may prove challenging in COVID-19 infections [30, 71]. The slightly immunocompromised condition of G6PD-deficient patients may prevent severe inflammation, which may be consequently beneficial to patients. However, it is important to note that this only applies to patients with less severe forms of G6PD deficiency; in severe class I G6PD deficiencies, G6PD levels may be too low to even adequately clear viral infections [72, 73]. However, the sample size of G6PD-deficient patients analyzed in the clinical study mentioned is too small to establish high study power. Additionally, the clinical presentation of G6PD-deficient patients may vary, so a well-designed large-scale clinical study or an animal model scalable to the human immune system would be required in order to provide sufficient evidence of this point.

Multiple articles and original research have concluded that oxidative stress plays a key role in COVID-19 and virally induced acute lung injuries [44, 74]. Exacerbation of acute lung injury via elevated ROS has been observed in animal models [74]. The reduction of oxidative stress has also been shown to inhibit viral replication [6, 41]. Herein, after reviewing existing data around ROS and viral infections, we suggest that the use of antioxidant or redox-modulating agents to control viral infection should be evaluated further. Particular attention should be paid to use in infections that could cause severe lung injury, including SARS-CoV-2. Polydatin, a specific redox-modulating agent, was promoted in existing literature, as it has potential to suppress oxidative inflammation induced by G6PD while working concurrently as an antioxidant [69]. Although the use of antioxidative agents like lipoic acid show a positive benefit at the cellular level, the results of cellular studies may not translate clinically due to the inherent complexities of human physiology [6, 41, 42]. Therefore, monitored clinical trials should be performed to examine whether adding antioxidative agents to standard treatment is safe and if doing so may improve the prognosis or reduce hospitalization in COVID-19 patients.

At present, clinical studies regarding the relationship between G6PD deficiency and COVID-19 infection are sparse. However, in vitro studies of the interaction between G6PD knocked-out cells and several viruses, including human coronavirus 229E, have been conducted; wild type and G6PD knocked-out cells were separately cultured with coronavirus 229E, and the number of viral genes was subsequently measured [6]. Similar studies could be performed with SARS-CoV-2 samples to evaluate whether G6PD knocked-out cells are more susceptible to SARS-CoV-2 infection at the cellular level. Additionally, further ex vivo studies could be done to assess the immune response against SARS-CoV-2 in G6PD-deficient animals. Clinical studies including the comparison of the SARS-CoV-2 viral load in infected G6PD-deficient patients versus normal patients could also be conducted. Such studies allow us to identify whether COVID-19 patients with G6PD deficiency are more contagious. Further testing of antioxidative agents on these models would help to evaluate the safety and effectiveness of such treatment. Though there is no guarantee that the actual host immune response will be analogous to that of the models, the suggested studies will allow us to have an overview of what could potentially happen in G6PD-deficient patients during SARS-CoV-2 infection; these results could serve as a foundation for future clinical studies [75]. Moreover, the physiological reaction of G6PD-deficient patients treated with remdesivir should be analyzed as to its use in treating COVID-19 patients expands [76]. When considering the findings of these studies and the potential of future research, physicians should be well-informed of the G6PD status of their COVID-19 infected patients, using remdesivir or other antiviral medication with great caution in positive patients, particularly those within class I [77].

Juneja et al. demonstrated that advanced age, male gender, diabetes, and abnormal hematological profile are associated with moderate to severe course of COVID-19 infection in a general population [78]. Given currently inadequate evidence to support that COVID-19 patients with G6PD deficiency have a worse prognosis in terms of mortality, severity, and rate of hospitalization [11], a larger scale study should be performed that encompasses different ages, ethnicity, gender, and more importantly the disease-alleles (hemizygous, homozygous or heterozygous) with consideration of G6PD deficiency variants. Furthermore, the incidence of G6PD-deficient individuals with SARS-CoV-2 infections should also be calculated; the viral load could also be compared between patients with wild-type G6PD and G6PD deficiency. The result of such studies will aid in defining whether G6PD deficiency is one of the factors of infection risk and whether such patients may experience heightened contagiousness when compared with unaffected individuals.

In conclusion, patients with G6PD deficiency are notorious for elevated levels of ROS in response to classic triggers including viral infections such as COVID-19. The slightly immunocompromised status of these patients is shown to favor viral replication; this may potentially result in increased viral load and infectivity within affected patients. Additionally, due to inherent proclivity to hemolytic, thrombotic, and other medically threatening events, G6PD-deficient patients may be limited in treatment options available to them, particularly in the case of COVID-19 infection. Though more research is demanded on the topic, preliminary studies suggest that antioxidative therapy that reduces ROS levels in these patients could prove beneficial in the treatment of viral infections in G6PD-deficient individuals.

## Data Availability

Not applicable.
